# Pharmacological induction of ferritin prevents osteoblastic transformation of smooth muscle cells

**DOI:** 10.1111/jcmm.12682

**Published:** 2015-10-26

**Authors:** Gergely Becs, Abolfazl Zarjou, Anupam Agarwal, Katalin Éva Kovács, Ádám Becs, Mónika Nyitrai, Enikő Balogh, Emese Bányai, John W. Eaton, Paolo Arosio, Maura Poli, Viktória Jeney, József Balla, György Balla

**Affiliations:** ^1^ Faculty of MedicineDivision of NephrologyDepartment of Internal MedicineUniversity of DebrecenDebrecenHungary; ^2^ Division of NephrologyDepartment of MedicineNephrology Research and Training Center and Center for Free Radical BiologyUniversity of Alabama at BirminghamBirminghamALUSA; ^3^ Faculty of MedicineDivision of NeonatologyDepartment of PediatricsUniversity of DebrecenDebrecenHungary; ^4^ Molecular Targets ProgramJames Graham Brown Cancer CenterUniversity of LouisvilleLouisvilleKYUSA; ^5^ Department of Molecular and Translational MedicineUniversity of BresciaBresciaItaly; ^6^ MTA‐DE Vascular Biology, Thrombosis and Hemostasis Research GroupHungarian Academy of SciencesDebrecenHungary

**Keywords:** ferritin, ferroxidase activity, β‐glycerophosphate, vascular calcification, vitamin D_3_

## Abstract

Vascular calcification is a frequent complication of atherosclerosis, diabetes and chronic kidney disease. In the latter group of patients, calcification is commonly seen in tunica media where smooth muscle cells (SMC) undergo osteoblastic transformation. Risk factors such as elevated phosphorus levels and vitamin D_3_ analogues have been identified. In the light of earlier observations by our group and others, we sought to inhibit SMC calcification *via* induction of ferritin. Human aortic SMC were cultured using β‐glycerophosphate with activated vitamin D_3_, or inorganic phosphate with calcium, and induction of alkaline phosphatase (ALP) and osteocalcin as well as accumulation of calcium were used to monitor osteoblastic transformation. In addition, to examine the role of vitamin D_3_ analogues, plasma samples from patients on haemodialysis who had received calcitriol or paricalcitol were tested for their tendency to induce calcification of SMC. Addition of exogenous ferritin mitigates the transformation of SMC into osteoblast‐like cells. Importantly, pharmacological induction of heavy chain ferritin by 3H‐1,2‐Dithiole‐3‐thione was able to inhibit the SMC transition into osteoblast‐like cells and calcification of extracellular matrix. Plasma samples collected from patients after the administration of activated vitamin D_3_ caused significantly increased ALP activity in SMC compared to the samples drawn prior to activated vitamin D_3_ and here, again induction of ferritin diminished the osteoblastic transformation. Our data suggests that pharmacological induction of ferritin prevents osteoblastic transformation of SMC. Hence, utilization of such agents that will cause enhanced ferritin synthesis may have important clinical applications in prevention of vascular calcification.

## Introduction

Cardiovascular disease remains the major cause of mortality in patients with chronic kidney disease (CKD) 1, 2, 3. In this regard, vascular calcification continues to be a significant clinical challenge with detrimental consequences and its prevalence and progression is rapidly accelerated once patients require renal replacement therapy 4. Two distinct forms of vascular calcification have been identified: intimal and medial calcification. The intimal calcification is commonly seen in atherosclerotic lesions, whereas calcification of the tunica media, also known as Mönckeberg's sclerosis, frequently accompanies ageing, diabetes mellitus and advanced CKD 5. The medial calcification was once thought to be a passive and benign finding, however, a strong body of evidence now suggests that medial calcification is a major cause of mortality and morbidity as it causes vascular stiffness, increases afterload with subsequent hypertrophy of the left ventricle and compromise of coronary perfusion 6. Moreover, the process of calcification of the vascular tree is now proven to be a delicate and regulated cellular process that entails transdifferentiation of SMC into osteoblast‐like cells. One of the markers of this transition is up‐regulation of ALP 7, 8. Alkaline phosphatase is a hydrolase enzyme that is important in early osteogenesis and has been demonstrated to hydrolyse and reduce the levels of a key inhibitor of hydroxyapatite formation namely, extracellular pyrophosphate. Given its function and the fact that its induction has been shown to be a major determinant of osteoblastic differentiation of SMC, ALP expression is commonly used as a surrogate to monitor the initiation and degree of vascular calcification 8. Transition of SMC into osteoblast‐like cells is also indicated by the increase in expression of osteocalcin, a major non‐collagenous protein found in bone matrix which is believed to regulate mineralization 7. Several promoters and inhibitors are now identified that are actively involved in commencing and propagation of vascular calcification 9. One of the most potent recognized inducers of vascular mineralization is elevated phosphorus (Pi) level. There is compelling evidence from clinical, animal and *in vitro* studies that hyperphosphataemia is a significant risk factor for the development of vascular calcification in CKD patients 10, 11, 12, 13. Another culprit that has been shown to induce calcification of SMC is excess of activated form of vitamin D although different forms of vitamin D have been shown to have different degree of effects on vascular calcification 14, 15.

Previously, we investigated whether induction of haeme oxygenase‐1/ferritin system alters high Pi‐induced SMC calcification *in vitro*. We found that iron (regardless of its ferric or ferrous state) released from the haeme moiety attenuates calcium deposition of SMC in a dose‐responsive manner when SMC are cultured in the presence of high Pi 16. Further studies revealed that iron‐induced ferritin heavy chain (FtH) expression was responsible for the observed inhibitory effects on calcium deposition. This notion was corroborated by utilizing recombinant FtH protein and its mutant form 16, 17. We found that while addition of FtH (devoid of iron) was able to prevent mineralization, the mutant form that lacks ferroxidase activity was ineffectual.

Severe decline in renal function and renal replacement therapy requirement are commonly associated with not only hyperphosphataemia but also iron deficiency 18. Major contributing factors to such iron deficiency include blood loss during haemodialysis, bleeding of cannula puncture sites following haemodialysis and too frequent diagnostic blood tests. Moreover, during chronic inflammatory diseases (such as CKD) there is an overproduction of a number of cytokines that exert multiple functions including increased production of hepcidin that in turn leads to the degradation of ferroportin (membrane iron transporter) 19. The overall effect translates to a ‘functional iron deficiency’ state which subsequently leads to decreased intracellular ferritin levels. Therefore, based on our previous studies we concluded that iron deficiency and the ensuing decrement in intracellular FtH expression facilitates vascular calcification in this group of patients and it has been suggested that overexpression of FtH would have inhibitory properties against mineralization of the vasculature in advanced CKD. However, based on iron's ability to readily accept or donate electrons, it is well known that iron overload and saturation of its sequestration threshold could lead to the generation of reactive oxygen species and consequent injurious effects 20. Hence, we sought to investigate whether 1,2‐dithiole‐3‐thione (D3T), a potent inhibitor of chemical‐induced tumours and a strong inducer of ferritin 21, 22, could be used to prevent calcification and osteoblastic differentiation of SMC. 1,2‐dithiole‐3‐thione has been shown to induce ferritin expression irrespective of iron concentrations *via* transcriptional mechanism that is mediated by the FtH electrophile/antioxidant responsive element 22.

To examine this hypothesis we used human aortic SMC and induced calcification by utilizing β‐glycerophosphate (BGP) with vitamin D_3_ analogues, as well as Pi with calcium.

## Materials and Methods

### Reagents

Calcitriol for *in vitro* experiments was purchased from Cayman Chemicals (Ann Arbor, MI, USA) and fetal bovine serum (FBS) from Life Technologies (Vienna, Austria). Recombinant ferritin subunits: H, L, H222 mutant were provided by Dr. Paolo Arosio (Department M.I. and Biomedical Technologies, University of Brescia, Brescia, Italy). Protease inhibitor tablets were from Roche (Mannheim, Germany). Paricalcitol (Zemplar^®^) and calcitriol (Calcijex^®^) for *in vivo* experiments were purchased from commercial source. Unless otherwise mentioned, all other reagents were purchased from Sigma‐Aldrich.

### Cell culture

Human aortic SMC were obtained from Cell Applications (San Diego, CA, USA), Lonza (Allendale, NJ, USA), Cambrex (Wokingham, UK). We used human SMC from one to five independent donors for our experiments. We designated the number of independent donors used for each figures that were more than one. Cells were grown in high glucose DMEM containing 15% FBS, 1 mM sodium pyruvate, 100 U/ml penicillin, 100 μg/ml streptomycin and neomycin (growth medium, GM). Cells were grown to confluence and used from passages 5 to 7. Media were changed every 2 days.

### Alkaline phosphatase activity assay

Cells were grown on twelve‐well plates for 7 days. Cells were washed with PBS twice and then solubilized with solubilization buffer [1% Triton‐X 100, 0.5% Igepal CA‐630, 1% protease inhibitor, 150 mM NaCl, 5 mM ethylenediaminetetraacetic acid (EDTA), 10 mM Tris]. The assay was performed by adding 35 μl sample to 130 μl of ALP Yellow Liquid Substrate and the kinetics of p‐nitrophenol formation was followed up for 30 min. at 405 nm incubating at 37°C. Maximum slope of the kinetic curves was used for calculation and the results were normalized to protein content.

### Alkaline phosphatase activity staining

Cells were grown on 48‐well plates for 7 days. Staining was done using the 85L3R kit from Sigma‐Aldrich. Briefly, cells were washed with PBS twice and fixed with citrate–acetone (2:3) solution. After fixation, cells were washed with ddH_2_O twice. Staining step was performed with Fast violet B salt containing 4% naphtol AS‐MX for 30 min. in dark. After staining, cells were washed again with ddH_2_O and images were obtained.

### Quantification of calcium deposition

Cells grown on 48‐well plates were washed twice with PBS and decalcified with 0.6 mol/l HCl for 24 hrs at 37°C. Calcium content of the supernatants was determined by the QuantiChrome Calcium Assay Kit (Gentaur, Brussels, Belgium). After decalcification, cells were solubilized with a solution of NaOH 0.1 mol/l and SDS 0.1%, and protein content of samples was measured with (bicinchoninic acid) BCA protein assay kit (Pierce, Rockford, IL, USA). Calcium content of the cells was normalized to protein content and expressed as μg/mg protein. Mineralization was also determined by Alizarin Red staining. Briefly, cells grown on 48‐well cell culture plates were washed with PBS and fixed with 4% paraformaldehyde. Plates were incubated for 10 min. in RT. Cells were washed with PBS and then stained with 2% Alizarin Red solution for 2 min. After the incubation cells were washed three times with distilled water and photos were taken under light microscope.

### Quantification of osteocalcin

Osteocalcin was detected from the extracellular matrix of cells grown on six‐well plates and was dissolved in 300 μl of EDTA [0.5 mol/l (pH 6.9)]. Osteocalcin content of the EDTA‐solubilized extracellular matrix samples was quantified by ELISA method (Bender MedSystems, Burlingame, CA, USA).

### Western Blot for ALP and FtH

Western blotting was performed with 10% SDS‐PAGE gels and 0.45 μm pore size nitrocellulose membrane (Amersham Biosciences, Little Chalfont, UK). Alkaline phosphatase was detected with a rabbit polyclonal antibody at 1:200 dilution (sc‐30203; Santa Cruz Biotechnology, Dallas, TX, USA) followed by HRP‐labelled anti‐rabbit IgG secondary antibody 1:15,000 (Amersham Biosciences). Ferritin heavy chain was detected using primary antibody against human FtH at 1:400 dilution (sc‐25617; Santa Cruz Biotechnology) and secondary antimouse IgG antibody (Amersham Biosciences) was used at 1:15,000 dilution. Antigen–antibody complexes were visualized with the horseradish peroxidase chemiluminescence system (Amersham Biosciences). Membranes were reprobed for glyceraldehyde‐3‐phosphate dehydrogenase (GAPDH). Mouse anti‐GAPDH (NB300‐221; Novus Biologicals, Littleton, CO, USA) at 1:1000 and secondary antimouse IgG (Amersham Biosciences) at 1:15,000 dilution were used.

### Quantitative reverse transcription‐polymerase chain reaction

Cells were grown on six‐well plates and total RNA was isolated with RNA‐STAT60 (Tel‐Test Inc, Friendswood TX, USA). SuperScript III reverse transcriptase kit (Invitrogen, Carlsbad, CA, USA) with oligodT (Promega, Madison, WI, USA) was used for reverse transcription. Alkaline phosphatase mRNA expression was determined by multiplex TaqMan Gene Expression Assays and was normalized to GAPDH (ALP: Hs00758162_m1; GAPDH: Hs99999905_m1; Applied Biosystem, Foster City, CA, USA). A total of 20 μl of reaction mixture contained 10 μl TaqMan Gene Expression Mastermix (Applied Biosystem) and 1 μl of assays from target and housekeeping gene and was completed with 8 μl of depc‐treated water. Reverse transcriptions and qPCRs were carried out using the C1000 Thermal Cycler with CFX 96 Real Time PCR System (Bio‐Rad, Hercules, CA, USA).

### FtH siRNA transfection

Small interfering RNA specific to FtH and negative control siRNA were obtained from Ambion (Austin, TX, USA). Smooth muscle cell transfection with siRNA was achieved using the Oligofectamine Reagent (Invitrogen). Briefly, the cells were plated overnight to form 60–70% confluent monolayers. Ferritin heavy chain siRNA at 30 nmol/l and transfection reagent complex were added to the cells in serum‐free medium for 4 hrs. Fresh normal GM was later added and the cells were incubated for another 20 hrs.

### *In vivo* study with different vitamin D receptor (VDR) activators

This study included five patients with end‐stage renal disease (ESRD) who receive intermittent haemodialysis on a regular schedule three times a week and suffer from secondary hyperparathyroidism. All patients met the criteria for paricalcitol and calcitriol medication as suggested by the guidelines of the Hungarian Society of Nephrology. Anticoagulated blood samples were collected after the routine dialysis before and 10 min. after the administration of drugs. The blood samples were centrifuged at 2000 × g for 10 min. Plasma of the blood samples were collected and kept frozen until the time of experiments when they were added to the medium. Specifically cells were treated by a 50% ratio of patient's plasma and 50% of growing media without FBS for 5 days. Samples collected after the drug administration were used with or without addition of apoferritin for assessing ALP activity, thus exposure of SMC of apoferritin lasted 5 days as well. The study design and patient care were reviewed and permission was obtained by the Ethics Committee of the University of Debrecen and the Hungarian Government.

### Statistical analysis

Statistical analysis was performed with GraphPad Prism 5 and by one‐way anova test followed by post hoc Bonferroni's Multiple Comparison test. A significant value of *P* < 0.05 was marked with *, *P* < 0.01 with ** and *P* < 0.001 was marked with ***. Non‐significant (ns) differences were also marked. Data are shown as mean ± SD.

Statistical analysis for Fig. 9C was performed employing Statistica for Windows. The variables were characterized by descriptive analyses (case number, median, quartiles). After comparing the results with Friedman anova, we used Wilcoxon matched pair test with Bonferroni correction to collate the paired samples. *P* ≤ 0.05 was considered significant.

## Results

### β‐glycerophosphate and activated vitamin D_3_ promote osteoblast‐like transformation of vascular SMC

**Figure 1:**
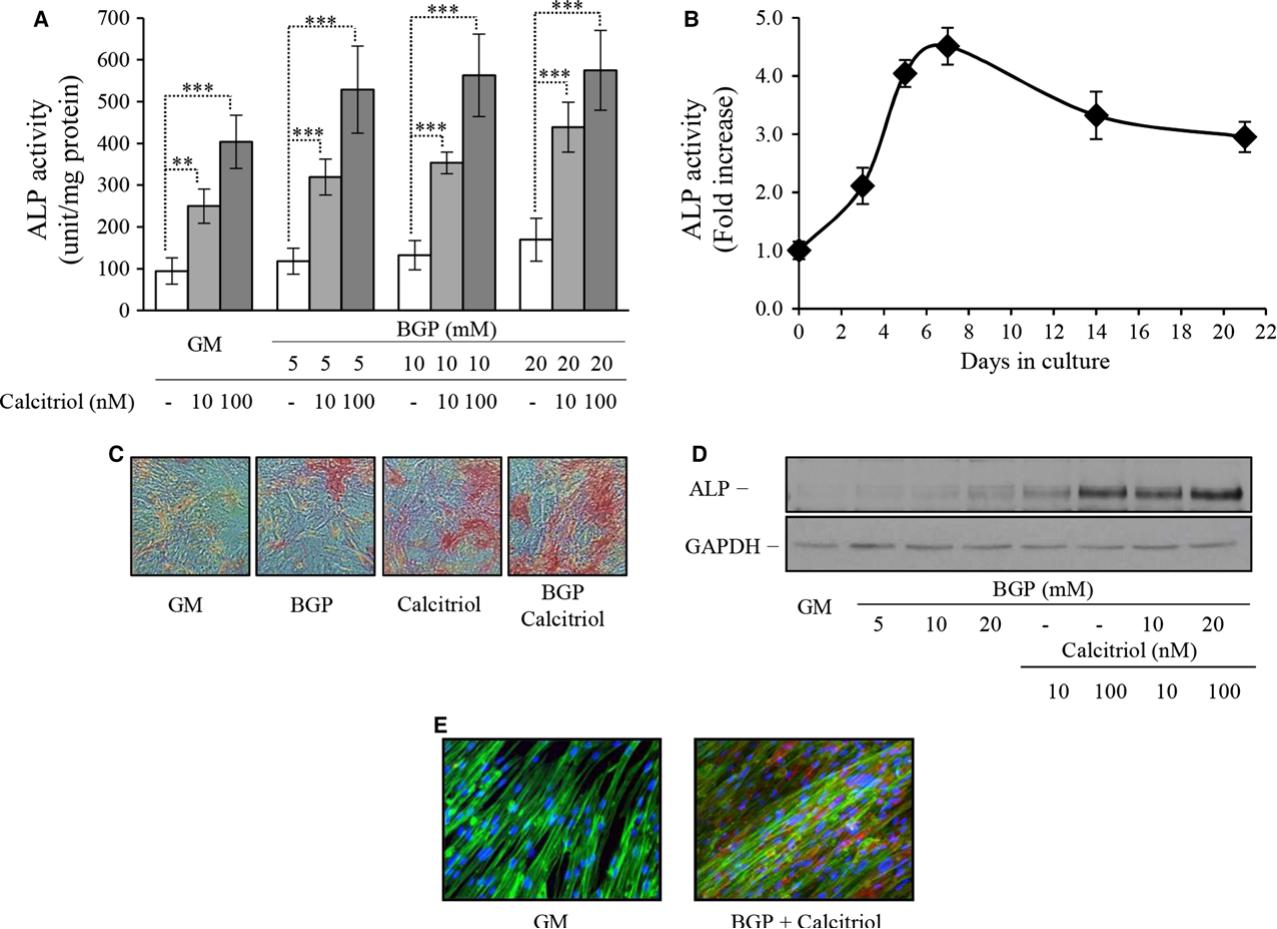
β‐glycerophosphate (BGP) and activated vitamin D_3_ (calcitriol) induces the osteoblastic transformation of human aortic smooth muscle cells (SMC). Human aortic SMC were cultured in growth medium (GM) or in the presence of increasing dose of BGP (5–10–20 mmol/l) with or without low and high dose of calcitriol (10–100 nmol/l) for 7 days. (**A**) Alkaline phosphatase enzyme activity was measured, and the results were normalized by protein content and data show the average of three separate experiments performed in duplicate from three independent donors. **P < 0.01, ***P < 0.001. (**B**) Cells from five independent donors were exposed to BGP with calcitriol for up to 3 weeks and enzyme activity was determined at 3, 5, 7, 14 and 21 days. (**C**) SMC were stained for alkaline phosphatase enzyme activity directly, and representative parts of the slides were selected, magnification 100×. (**D**) Alkaline phosphatase enzyme level was detected by Western Blot, where loading control was performed by GAPDH. (**E**) Alkaline phosphatase induction was visualized by immunofluorescence staining. Blue colour represents the nuclei, green is for the fibrillar actin and red for the alkaline phosphatase, magnification 400×.

Our first goal was to test the osteoblast‐like transformation of human aortic SMC induced by BGP and activated vitamin D_3_ (Calcitriol). Both inducers have the ability to increase ALP activity in dose‐dependent manner. As expected we found that combined treatment resulted in higher enzyme activity of ALP (Fig. 1A). The induction of enzyme activity was time‐dependent leading to a pronounced elevation at 7 days of exposure (Fig. 1B). Therefore, in our studies the expression of ALP in human SMC was assessed at 7 days. Staining for ALP activity showed increased number of positively stained cells following treatment with BGP or calcitriol alone or together (Fig. 1C). Western blots confirmed that the increased activity was because of the increment of ALP expression. β‐glycerophosphate and calcitriol increased the enzyme level dose‐dependently and the additive effect is also evident in this representative experiment (Fig. 1D). Expression of ALP protein was also estimated using immunofluorescence staining. Untreated cells were almost completely negative with this staining, whereas cells exposed to BGP and/or calcitriol show increased fluorescence signal indicating higher expression. The cytoskeleton was counterstained for fibrillar actin (Fig. 1E).

### Ferritin mitigates induction of ALP activity

**Figure 2:**
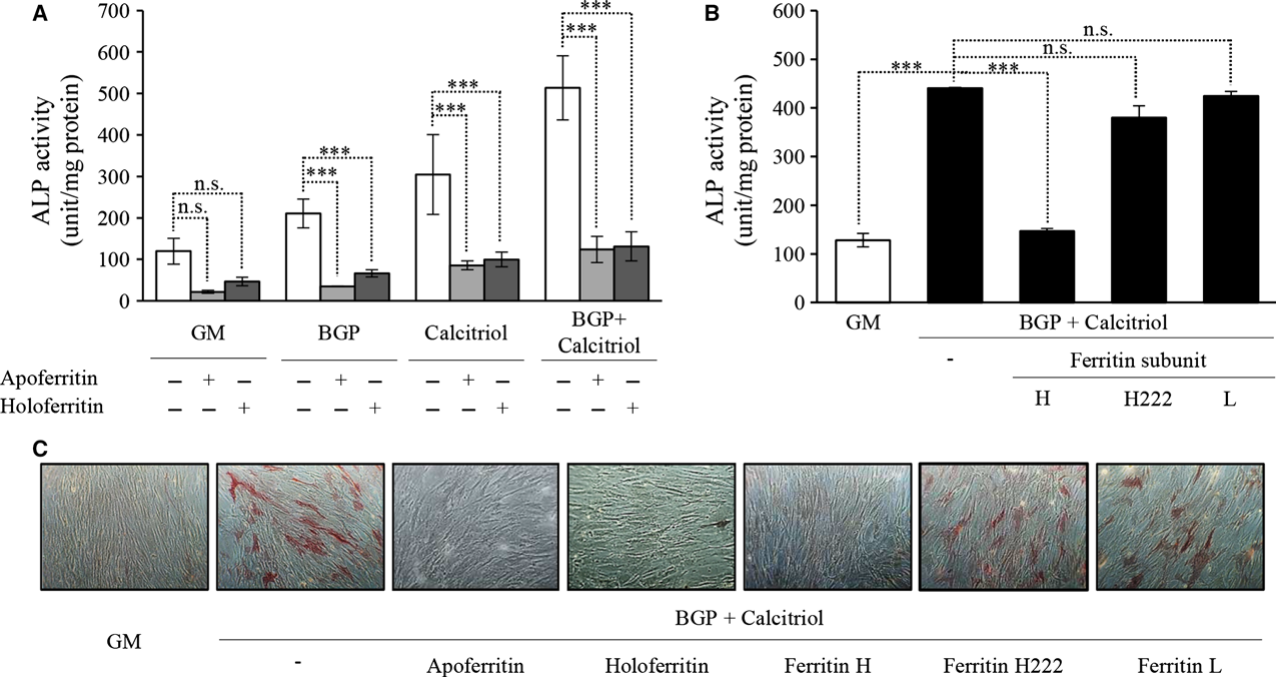
Ferritin inhibits the osteoblastic transformation of human aortic smooth muscle cells (SMC) induced by β‐glycerophosphate (BGP) and activated vitamin D_3_ (calcitriol). (**A**) SMC were cultured in growth media (GM) or in the presence of BGP (10 mmol/l) with or without calcitriol (10 nmol/l) with additional apo‐ or holoferritin (2 mg/ml) for 7 days. (**B**) SMC were cultured in GM or in the presence of BGP (10 mmol/l) and calcitriol (10 nmol/l) with different subunits of ferritin molecule, H‐, L‐ and mutant H‐chain respectively. After 7 days, alkaline phosphatase enzyme activity was measured, and the results were normalized by protein content and data show the average of three separate experiments performed in duplicate. ***P < 0.001. (**C**) SMC were cultured in GM or in the presence of BGP (10 mmol/l) and calcitriol (10 nmol/l) with additional apo‐ or holoferritin or different subunits of ferritin molecule, H‐, L‐ and mutant H‐chain respectively for 7 days, and cells were stained for alkaline phosphatase enzyme activity directly, and representative parts of the slides were selected, magnification 100×.

Human aortic SMC were treated with BGP and calcitriol alone or in combination to investigate the induction of ALP in the presence or absence of apo‐ and holoferritin. Both forms of ferritin decreased the activity of ALP and our findings show that there were no significant differences between the two forms (Fig. 2A). Based on this result that elaborates both forms of ferritin are capable of marked inhibition of ALP expression induced either by BGP or activated vitamin D_3_, the subsequent experiments were performed with the combination of both BPG and vitamin D_3_.

As ferritin shell is made of both the H and L chains and to delineate the individual effects of each form of ferritin, we performed the following experiment using H‐ (FtH) and L‐ recombinant ferritin. Furthermore, we also utilized the H222 mutant form of FtH which is identical to the FtH, but lacks ferroxidase activity based on the mutation 23. Alkaline phosphatase activity assays showed that inhibition of transformation only occurred when cells were pre‐incubated with FtH chain, whereas L chain and H222 (both of which lack ferroxidase activity) had no effect (Fig. 2B).

Alkaline phosphatase staining of treated cells showed increased number of positively stained cells treated with both inducing agents *versus* untreated cells. Addition of either apo‐ or holoferritin led to significant inhibition of ALP induction. In addition, ferritin subunits were also used in staining assays to show the inhibitory effect of ferroxidase activity. Inhibition of transformation only occurred when cells were pre‐incubated with ferroxidase‐active H chain (Fig. 2C).

**Figure 3:**
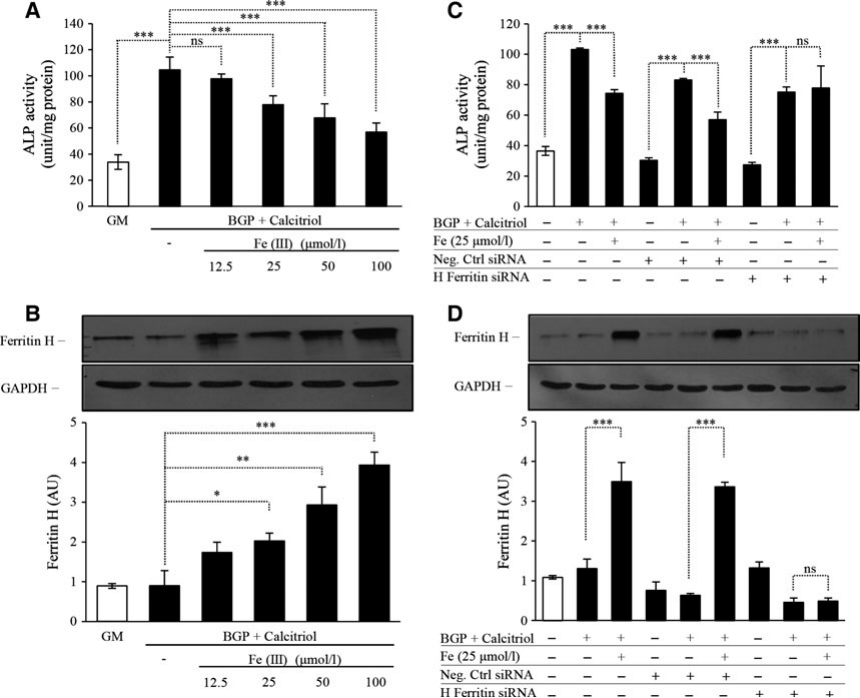
Exogenous iron inhibits the osteoblastic transformation of human aortic smooth muscle cells (SMC) induced by β‐glycerophosphate (BGP) and activated vitamin D_3_ (calcitriol) by increased ferritin expression. SMC were cultured in growth medium (GM) or in the presence of BGP (10 mmol/l) and calcitriol (10 nmol/l) with increasing dose of ferric‐(III)‐ammonium citrate (12.5–25–50–100 μmol/l) for 7 days. (**A**) Alkaline phosphatase enzyme activity was measured, and the results were normalized by protein content and data show the average of three separate experiments performed in duplicate. ***P < 0.001. (B) Ferritin H‐chain expression was detected by Western blot as shown in upper panel, and after densitometry the result was normalized to GAPDH, shown in lower panel. *P < 0.05, **P < 0.01, ***P < 0.001 (C) SMC were cultured in GM or in the presence of BGP (10 mmol/l) and calcitriol (10 nmol/l) with additional ferric‐(III)‐ammonium citrate (25 μmol/l) for 5 days with or without negative control (NC) or H‐ferritin (FtH)‐specific siRNA. Alkaline phosphatase enzyme activity was measured, and the results were normalized by protein content and data show the average of two separate experiments performed in duplicate. ***P < 0.001. (**D**) To evaluate the efficiency of ferritin H‐chain silencing protein expression was detected by Western blot shown in upper panel, and the results of densitometry after normalizing to GAPDH are shown in lower panel.

To determine whether the effects of exposure of cells to exogenous ferritins were specific for ferritin *per se* (as opposed to effects which might be mediated by the fluid phase endocytosis of exogenous ferritin), cells were also pre‐exposed to iron (in the form of ferric ammonium citrate) which induces ferritin synthesis by increasing intracellular iron (Fig. 3B). The treatment of cells with iron and subsequent induction of ferritin resulted in mitigation of BGP and calcitriol‐induced ALP induction (Fig. 3A).

The specific involvement of ferritin in the prevention of ALP induction is supported by experiments using siRNA. Again, we exposed cells to ferric ammonium citrate, but in this case, inhibited ferritin synthesis using anti‐FtH siRNA. The suppression of FtH induction abrogated the blockade of ALP induction which proceeded in cells which had not been pre‐treated with BGP and calcitriol (Fig. 3C). Therefore, these results demonstrate that FtH induction – irrespective of the mode and whether *via* fluid phase endocytosis of exogenous FtH or induction of FtH synthesis caused by exposure to additional iron in the culture medium – directly prevents ALP expression and activity which normally occurs under conditions favouring calcification. We also confirmed the efficiency of siRNA, and observed an approximately 80% decrease in FtH protein expression for up to 4 days after transfection (Fig. 3D).

**Figure 4:**
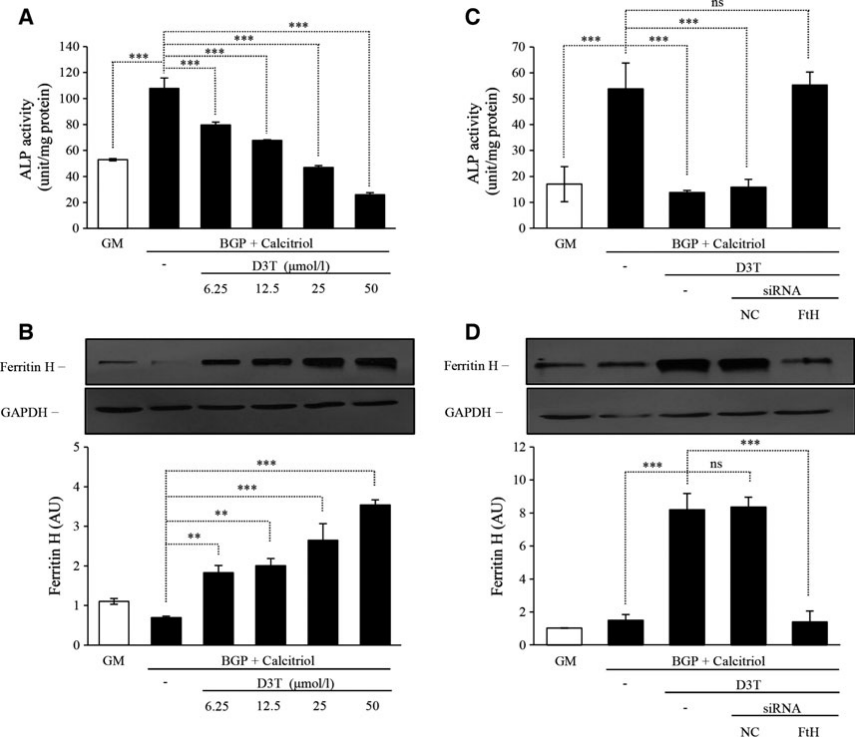
Pharmacological induction of ferritin inhibits the osteoblastic transformation of human aortic smooth muscle cells (SMC) induced by β‐glycerophosphate (BGP) and activated vitamin D_3_ (calcitriol). SMC were cultured in the growth medium (GM) or in the presence of BGP (10 mmol/l) and calcitriol (10 nmol/l) with increasing dose of 3H‐1,2‐Dithiole‐3‐thione (D3T) (6.25–12.5–25–50 μmol/l) for 7 days. (**A**) Alkaline phosphatase enzyme activity was measured, and the results were normalized by protein content and data show the average of three separate experiments performed in duplicate. ***P < 0.001. (**B**) Ferritin H‐chain expression was detected by Western blot as shown in upper panel, and after densitometry the result was normalized to GAPDH, shown in lower panel. **P < 0.01, ***P < 0.001 (**C**) SMC were cultured in GM or in the presence of BGP (10 mmol/l) and calcitriol (10 nmol/l) with additional D3T (50 μmol/l) for 4 days with or without negative control (NC) or H‐ferritin (FtH)‐specific siRNA. Alkaline phosphatase enzyme activity was measured, and the results were normalized by protein content and data show the average of two separate experiments performed in duplicate. ***P < 0.001. (**D**) To evaluate the efficiency of ferritin H‐chain silencing protein expression was detected by Western blot shown in upper panel, and the results of densitometry after normalizing to GAPDH are shown in lower panel.

Besides iron, which is the physiological inducer of ferritin, other agents could also be utilized to induce endogenous ferritin expression. We chose the 3H‐1,2‐Dithiole‐3‐thione (D3T). Human aortic SMC treated with increasing dose of D3T in the presence of BGP and calcitriol showed lower activity of ALP, and the decreased enzyme activity showed an inverse relationship with the increasing dosage of D3T (Fig. 4A). We confirmed induction of FtH as a dose‐dependent response to D3T administration (Fig. 4B). To demonstrate that FtH induction by D3T is responsible for the inhibitory effect on ALP expression in SMC, we transfected cells with siRNA for FtH and measured ALP enzyme activity. Indeed, ALP activity was not attenuated in D3T‐treated SMC grown in BGP and calcitriol containing media after silencing FtH (Fig. 4C). The efficiency of silencing FtH was approximately 70% (Fig. 4D) in our experiment.

### Elucidation of mechanism of inhibition of human aortic SMC differentiation to osteoblasts

**Figure 5:**
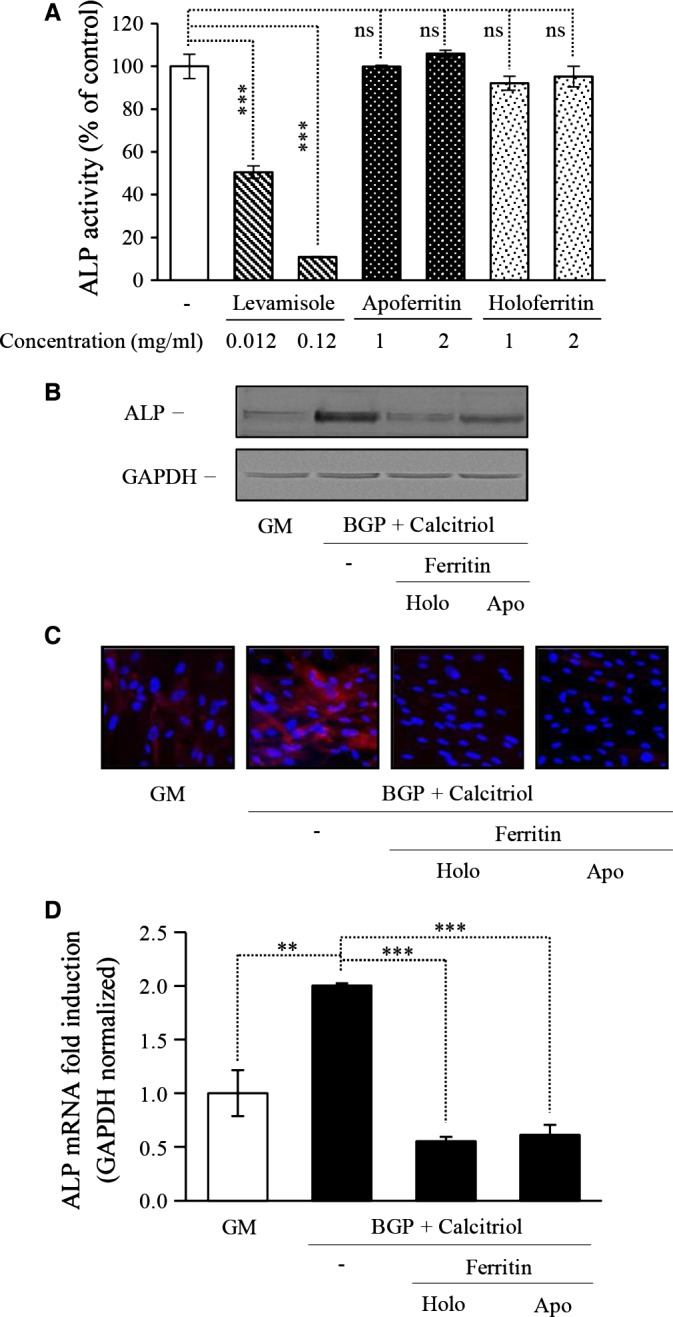
Elucidation of mechanism of inhibition of smooth muscle cells (SMC) differentiation to osteoblasts. (**A**) SMC were treated with β‐glycerophosphate (BGP) (10 mmol/l) and activated vitamin D_3_ (calcitriol) (10 nmol/l) for 7 days and the cell lysates was used for *in vitro* measurements. Apo‐ and holoferritin in addition to levimazole, a well‐known ALP inhibitor was used to evaluate the direct effects on enzyme activity. Enzyme activity was measured in duplicate, ***P < 0.001. SMC were cultured in growth medium (GM) or in the presence of BGP (10 mmol/l) and calcitriol (10 nmol/l) with additional apo‐ or holoferritin (2 mg/ml) for 7 days. (**B**) Alkaline phosphatase enzyme level was detected by Western Blot, where loading control was performed by GAPDH. (**C**) Alkaline phosphatase induction was visualized by immunofluorescence staining. Blue colour represents the nuclei and red colour shows the expression of alkaline phosphatase, magnification 400×. (**D**) Alkaline phosphatase mRNA expression was detected by RT‐PCR and results were normalized by GAPDH mRNA content and data show the average of two separate experiments performed in duplicate. **P < 0.01, ***P < 0.001.

We next sought to investigate the mechanism by which FtH provides inhibitions of ALP and hence osteoblastic transformation of SMC. *In vitro* measurement of enzyme activity was performed for which cells were treated with BGP and calcitriol for 7 days and then solubilized. Supernatant of cell lysates were exposed to the effect of apo‐ and holoferritin. Levamisole, a well‐known inhibitor of ALP was used to illustrate the inhibition. We found that such a treatment did not result in inhibition of ALP activity and hence results suggest thatferritin does not directly alter ALP enzyme activity (Fig. 5A).

We then examined protein expression by Western blot. Cells treated with calcifying media showed increased ALP expression, whereas both apo‐ and holoferritin diminished the induction (Fig. 5B). Moreover, we show that ferritin molecules can inhibit the increased level of ALP protein visualized with immunofluorescence staining (Fig. 5C).

Next, we examined the possibility of observed inhibitory effects at transcriptional level utilizing RT‐PCR. mRNA expressions of ALP in samples were measured and normalized by GAPDH and the fold increase to growth media was calculated. We show that calcifying condition resulted in up‐regulation of ALP mRNA. Results revealed that both apo‐ and holoferritin significantly decreased ALP mRNA levels (Fig. 5D). These results indicate that regulation of ALP expression in SMC by ferritin occurs at transcriptional level.

### Ferroxidase activity is the key inhibitory mediator of osteoblastic transformation

**Figure 6:**
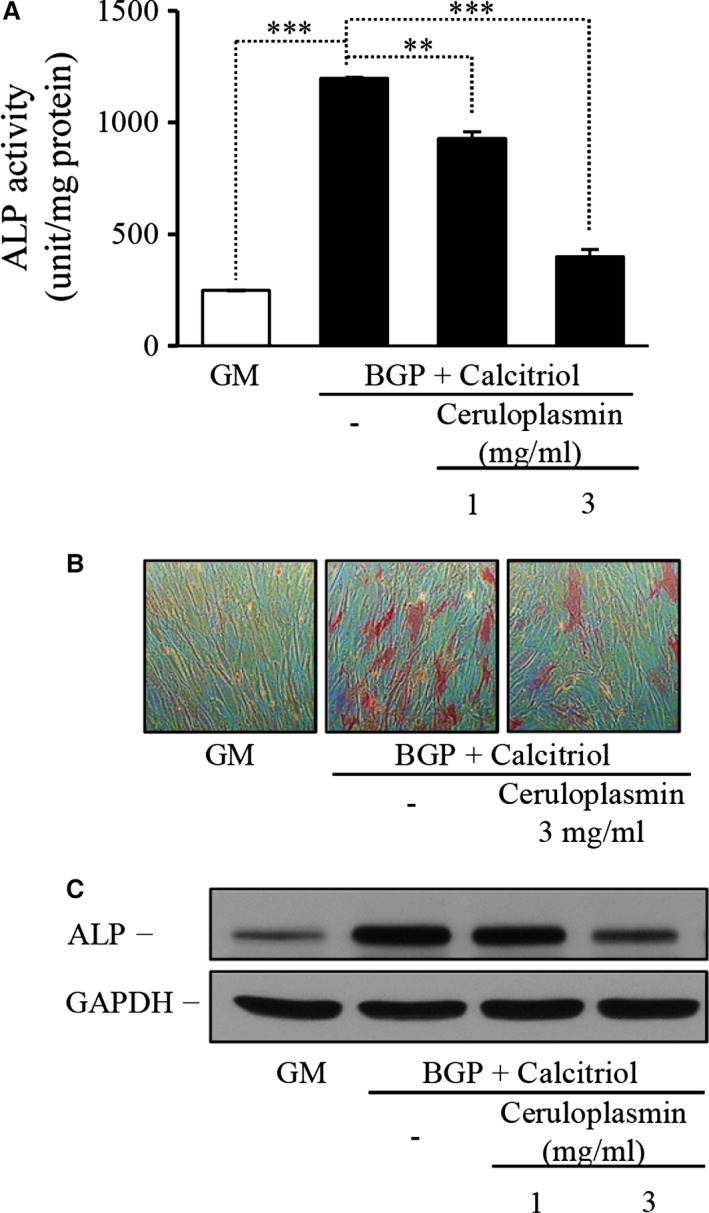
Ferroxidase activity of ceruloplasmin also inhibits the differentiation of smooth muscle cells (SMC) to osteoblasts. SMC were treated with β‐glycerophosphate (BGP) (10 mmol/l) and activated vitamin D_3_ (calcitriol) (10 nmol/l) in the presence of increasing dose of ceruloplasmin (1–3 mg/ml) for 7 days. (**A**) Alkaline phosphatase enzyme activity was measured, and the results were normalized by protein content and data show the average of two separate experiments performed in duplicate. **P < 0.01, ***P < 0.001. (**B**) Cells were stained for alkaline phosphatase enzyme activity, and representative parts of the slides were selected, magnification 100×. (**C**) Alkaline phosphatase enzyme level was detected by Western Blot, and GAPDH served as loading control.

Ceruloplasmin is a copper‐binding and carrier protein that is mainly produced by hepatocytes. However, for the purposes of our experiments it served as an important control arm as it, similar to FtH, possesses ferroxidase activity. Ferroxidase activity of ceruloplasmin dose‐dependently inhibited the ALP activity enhanced by osteoblastic inducers (Fig. 6A). Induction of ALP by calcitriol alone was also inhibited by ceruloplasmin by 70%. Staining experiments also demonstrated further evidence supporting the inhibitory role of ferroxidase activity (Fig. 6B). The decreased activity was caused by the lowered protein expression, which was detected by Western blot analysis (Fig. 6C).

### Inhibition of human aortic SMC mineralization by heavy chain ferritin

**Figure 7:**
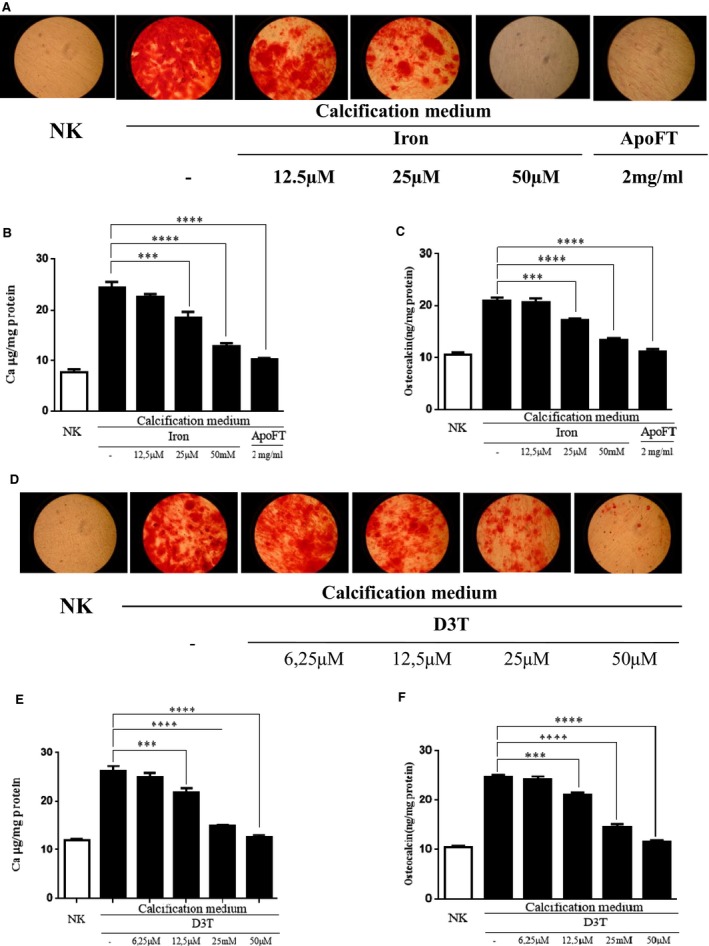
Mineralization of vascular smooth muscle cells induced by inorganic phosphate and calcium is inhibited by induction of ferritin. Calcification occured by supplementation of growth medium (GM) with 1.2 mmol/l inorganic phosphate and 0.9 mmol/l calcium chloride. (**A**) Alizarin Red staining of the cells shows the mineralization process and the inhibition by increasing dose of iron and apoferritin. (**B**) Iron inhibits extracellular calcium deposition of human aortic smooth muscle cells (SMC) in a dose‐dependent manner, where apoferritin also prevents mineralization. Calcium content of the cells was measured at 5 days and normalized by the protein content of the cells. (**C**) Iron and apoferritin attenuate up‐regulation of osteocalcin induced by calcifying condition. SMC were cultured in GM or in calcification medium with increasing dose of ferric‐(III)‐ammonium citrate (12.5–25–50 μmol/l) or 2 mg/ml apoferritin for 5 days. Data is derived from four separate experiments are shown as mean ± SD, **P < 0.01; ***P < 0.0001. (**D**) Representative images of SMC with Alizarin red staining indicating the inhibition of mineralization with increasing dose of 3H‐1,2‐Dithiole‐3‐thione (D3T). SMC were cultured in GM or in calcifying condition as described above or supplemented with 6.25, 12.5, 25 and 50 μmol/l D3T during 5 days. Exposure of SMC to D3T decreased the accumulation of calcium (**E**) and osteocalcin levels (**F**) in a dose‐responsive manner. Graphs are shown mean ± SD of three separate experiments. **P < 0.01; ***P < 0.001.[Correction added on 26 January 2016 after first online publication: Figure 7 was presented incorrectly and has been amended in this version.]

**Figure 8:**
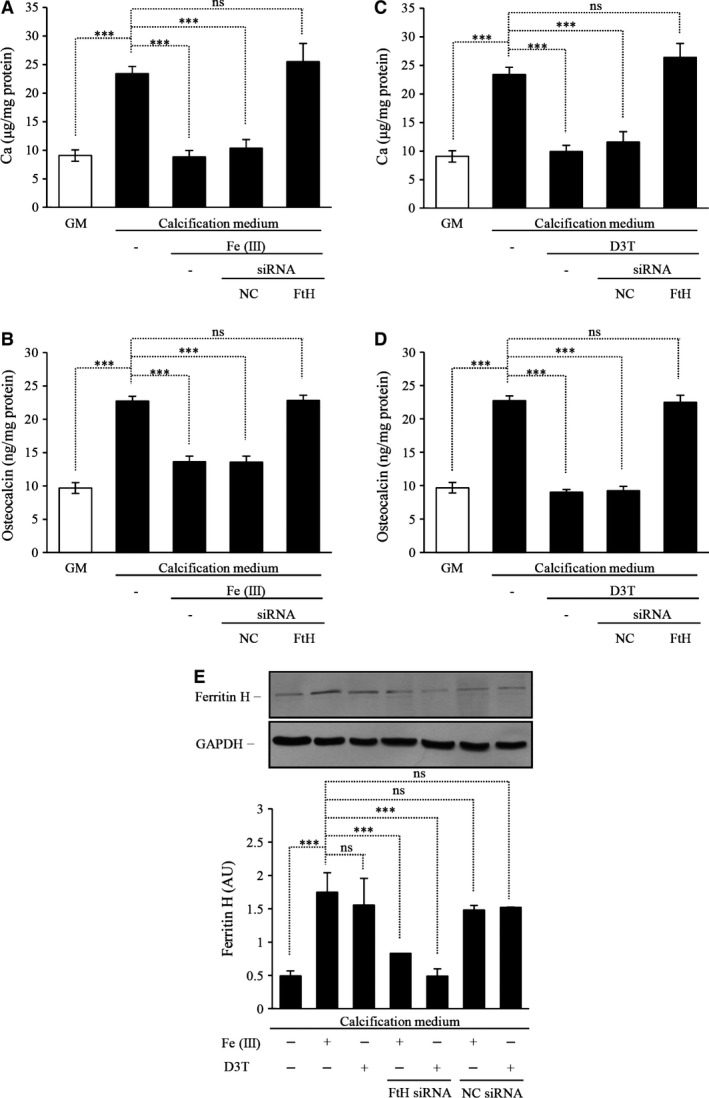
Ferritin heavy chain (FtH) is the main modulator of inhibition of mineralization of vascular smooth muscle cells. Smooth muscle cells (SMC) were transfected with siRNA for FtH or negative control siRNA (NC) 24‐hr before the experiments. Cells were cultured in growth media (GM) or calcifying media (additional 1.2 mmol/l inorganic phosphate and 0.9 mmol/l calcium to GM) alone or in the presence of 25 μmol/l ferric‐(III)‐ammonium citrate or 50 μmol/l D3T for 4 days. (**A**) Iron exposure after FtH silencing failed to attenuate calcium and (**B**) osteocalcin accumulation. (**C**) D3T treatment after silencing of FtH loses the capacity to inhibit the calcium deposition and (**D**) osteocalcin accumulation in extracellular matrix. (**E**) Western blot demonstrates the efficiency of FtH silencing. GAPDH served as loading control. Results are shown as mean values ± SD. ***P < 0.001.

Although the expression of ALP as an early marker of mineralization was increased after exposure of human SMC to BGP and calcitriol mineralization did not occur even if cells from five independent donors were cultured for up to 3 weeks. This is in accordance with the literature – bovine SMC are mostly employed in calcification studies as they are more ready to mineralize as compared to human SMC. To demonstrate that by up‐regulating ferritin by iron or D3T inhibits not only the expression of ALP, but it affects calcification as well in human SMC, we exposed human aortic SMC to 1.2 mmol/l Pi and 0.9 mmol/l calcium (calcification medium) and performed Alizarin red staining as well as measured the calcium accumulation. Granular deposits developed in cells grown in calcification medium, but not in the control culture grown in normal GM (Fig. 7A and D), as demonstrated by Alizarin red staining. We found that addition of iron or D3T to the calcification medium suppresses granular deposit development (Fig. 7A and D) and extracellular calcium deposition in a dose‐responsive manner (Fig. 7B and E), causing marked inhibition at concentrations of 25 and 50 μmol/l, respectively. As iron is a very potent inducer of ferritin and D3T also markedly enhances the intracellular ferritin content (Figs 3 and 4), next we tested whether the observed inhibitory effect of iron or D3T on calcium deposition is mimicked by apoferritin. We found that iron‐free apoferritin at a dose of 2 mg/ml abolishes granule formation (Fig. 7A) and inhibits calcium deposition (Fig. 7B). To confirm the inhibitory effect of iron or D3T on calcium accumulation occurs *via* FtH, we transfected human SMC with small interfering RNA specific to FtH. We observed an approximately 70% efficiency in FtH silencing (Fig. 8E). As we exposed cells to ferric ammonium citrate or D3T the suppression of FtH induction resulted in abrogation of the inhibition of calcium accumulation (Fig. 8A and C). This result indicates that the elevation of intracellular FtH by iron or D3T is the main mediator of inhibition of the calcification of extracellular matrix.

Next we investigated the presence of the bone‐specific protein, osteocalcin, in the extracellular matrix. Maintaining of human SMC in calcification medium for 7 days resulted in a significant increase in osteocalcin content compared to the control Figure 7C and F. Iron or D3T decreased the up‐regulation of osteocalcin in a dose‐responsive manner (Fig. 7C and F). In addition, apoferritin also abolished expression of osteocalcin (Fig. 7C). As we silenced FtH in human SMC treated with ferric ammonium citrate or D3T (Fig. 8E) the inhibition of osteocalcin accumulation in extracellular matrix was abrogated (Fig. 8B and D). Taken together, intracellular ferritin induction is a major inhibitory mediator of calcification and transdifferentiation of SMC.

### Vitamin D_3_ receptor agonist in the osteoblastic transformation

**Figure 9:**
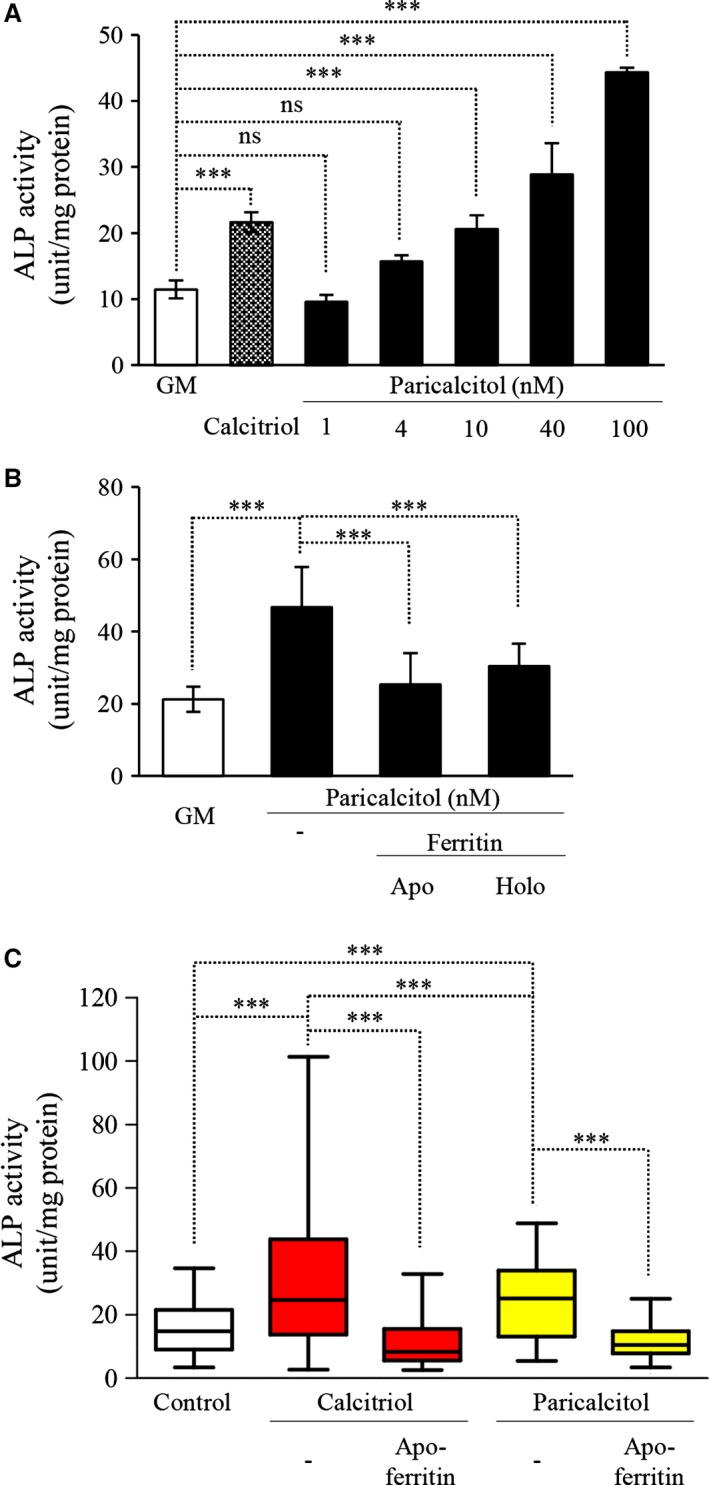
Vitamin D analogues increases the osteoblastic transformation of vascular smooth muscle cells (SMC). (**A**) SMC were cultured in growth medium (GM) or in the presence of activated vitamin D_3_ (calcitriol) (10 nmol/l) or with increasing dose of paricalcitol (1–4–10–40–100 nmol/l) for 7 days. Alkaline phosphatase enzyme activity was measured, and the results were normalized by protein content and data show the average of the triplicates, ***P < 0.001. (**B**) SMC were cultured in GM or in the presence of paricalcitol (40 nmol/l) with additional apo‐ or holoferritin (2 mg/ml) for 7 days. Enzyme activity was measured, and the results were normalized by protein content and data show the average of two separate experiments performed in duplicate, ***P < 0.001. (**C**) Plasma samples from volunteers from our dialysis centre were collected right after the haemodialysis session (control) and after the administration of calcitriol/paricalcitol. After the administration of drugs we waited 5 min. to reach a steady‐state blood concentration and then the samples were taken. SMC were treated with 1:1 ratio with serum‐free media and plasma samples for 5 days. Alkaline phosphatase activity was measured, and the results were normalized by protein content and as a result of the high variability of patients values data show maximum and minimum values besides of the median and interquartile range, experiments were performed in duplicate.

Paricalcitol was able to increase the activity of ALP in human aortic SMC and the inducing effect was dose‐dependent (Fig. 9A). Ferroxidase activity of apo‐ and holoferritin inhibited the increased ALP enzyme activity induced by paricalcitol (Fig. 9B).

### *In vivo* study with different VDR activators and ferritin

Plasma samples from patients with ESRD and secondary hyperparathyroidism who were treated with calcitriol or paricalcitol were collected and added to human aortic SMC. After 5 days, calcitriol significantly increased enzyme activity, and apoferritin abrogated this induction. Paricalcitol also significantly induced the activity of ALP albeit to a lesser degree. Again, addition of ferritin lowered ALP activity to baseline level irrespective of the inducer (Fig. 9C).

## Discussion

The findings presented in this study corroborate the inhibitory role of FtH in the inhibition of SMC mineralization and osteoblastic transformation. To induce mineralization we used BGP with or without the addition of calcitriol. These experiments confirmed previous reports identifying elevated Pi and high levels of activated vitamin‐D (calcitriol) as inducers of calcification of SMC 15, 24, 25. We used ALP expression as a surrogate of SMC transition to osteoblasts as previously reported 8. We validated that FtH prevents calcification and osteoblastic transformation of SMC, irrespective of the inducers used in this study. In this regard, ferroxidase activity was confirmed to be essential in the mitigation of calcification. We used D3T, a well‐known chemo‐preventive agent that is known to induce ferritin expression 21, 22.

**Figure 10:**
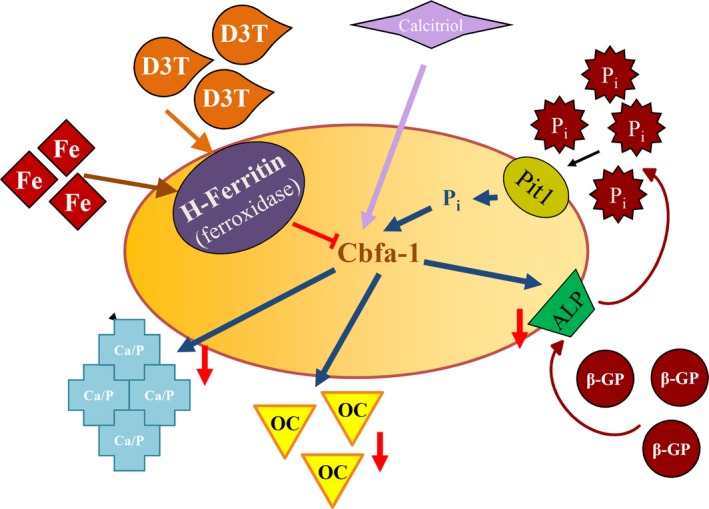
Suggested mechanism of how induction of ferritin *via* D3T prevents smooth muscle cells mineralization. Transition of vascular smooth muscle cells into osteoblast‐like cells can be triggered by phosphate [inorganic (Pi), β‐glycerophosphate (β‐GP)] or activated vitamin D_3_ (calcitriol). Pi taken up by sodium‐phosphorus co‐transporter‐1 (Pit‐1) activates core binding factor alpha‐1 (Cbfa‐1) responsible for the expression of osteoblast‐specific genes. Induction of ferritin heavy chain with ferroxidase activity by 3H‐1,2‐Dithiole‐3‐thione (D3T) or iron abrogates the elevation of osteoblast‐specific genes [transcription factor Cbfa‐1, osteocalcin (OC), alkaline phosphatase (ALP)] and the accumulation of calcium in the extracellular matrix.

We demonstrate that induction of ferritin *via* D3T abrogates SMC transition to osteoblasts and therefore prevents calcification of extracellular matrix (Fig. 10). Furthermore, we found that culture of SMC with serum samples from ESRD patients receiving calcitriol or paricalcitol leads to osteoblastic transition and we also show that addition of FtH prevents such transition.

Cardiovascular disease remains the leading cause of death in patients with advanced CKD and ESRD 2, 26, 27. Evidence suggests that adjusted cardiovascular attributed mortality is higher by about 10–20‐fold in CKD patients compared to the general population 28. The increased prevalence of cardiovascular disease in this group of patients is, at least in part, attributable to vascular calcification 29. Increased prevalence of this condition in the younger patient population requiring renal replacement therapy is a strong confirmation that the uraemic milieu generates the perfect storm for initiation and acceleration of vascular calcification 30, 31. Increased Pi level is a significant risk factor and has been confirmed in multiple studies to be a key regulator of vascular calcification 32. Other inducers of vascular calcification have also been identified 33. The vitamin D receptor analogues have been shown to induce mineralization of the vascular tree as well. However, different analogues have been shown to exert variable results 4, 14. Importantly, calcitriol and its analogues are routinely used to manage secondary hyperparathyroidsm that is a frequent complication of end‐stage kidney disease. The SMC are known to express vitamin‐D receptor that is up‐regulated by calcitriol. Furthermore, previous studies indicate that calcitriol can exert multiple effects on the proliferation and differentiation of SMC. In addition, it has been demonstrated that supraphysiological doses of calcitriol can induce vascular calcification in both *in vitro* and animal models 34. It is suggested that calcitriol increases SMC mineralization by increasing the RANKL/osteoprotegerin ratio 14. Based on the above mentioned evidence we used Pi and calcitriol to induce osteoblast transition of SMC.

Ferritin is an ancient, large, spherical protein with highly conserved three‐dimensional structure and is the most important intracellular iron handling machinery 35, 36. The ferritins are a family of proteins similar to spherical shells, designed to sequester and store large amounts of iron in a safe, soluble and bioavailable form. Ferritin is made of 24 subunits of two types (H, heavy and L, light chain) whose proportion depends on the iron status of the cell, the tissue and the organ. The two ferritin polypeptides are related, but FtH carries a ferroxidase activity to oxidize Fe^2+^ to Fe^3+^ allowing safe incorporation of iron into the shell. Ferritin shells can store up to 4500 iron atoms. Ferritin acts as a depot, sequestering excess iron and allowing for the mobilization of iron when needed 35, 36. Our understanding of ferritin and its functions has markedly enhanced in the past decade and novel functions other than regulation of iron metabolism and homoeostasis are now attributed to ferritin 16, 37, 38, 39, 40, 41.

Previously, we reported that haeme and iron‐mediated induction of FtH prevents calcification and osteoblastic differentiation of SMC induced by high Pi 16. We reported that ferroxidase activity of FtH was the key mediator of the observed inhibitory effects 16. Importantly, we previously found that the induction of FtH was able to actively prevent the process of transformation of SMC into osteoblast‐like cells and also inhibit osteoblast activity *via* down‐regulation of Cbfa‐1 16, 40, the main transcriptional factor mediating osteogenesis and responsible for the induction of osteoblast‐specific gene expression 42. Furthermore, ferritin did not affect Pi uptake *via* Pit1 (sodium‐phosphorus co‐transporter) 16. These findings were the first report to suggest a relationship between deranged iron metabolism that is a frequent complication of CKD and vascular calcification. Patients who are dependent on renal replacement therapy are susceptible to iron deficiency for a multitude of reasons that include frequent blood loss during haemodialysis and frequent diagnostic testing 43. Moreover, increased expression and secretion of hepcidin in chronic inflammatory states such as CKD translates to internalization and degradation of ferroportin in lysosomes 44, 45. The loss of ferroportin from cell membrane consequently ablates cellular iron export. Evidence suggests that increased hepcidin levels can lead to functional iron deficiency and subsequently anaemia by interrupting intestinal iron absorption and inducing the retention of iron within recycling reticuloendothelial macrophages. Therefore, it is evident that CKD patients are at increased risk of developing iron scarcity ensued by significant decrement in intracellular ferritin expression. We suggested that such decrement exacerbates SMC transition to osteoblasts in the uraemic environment. We were hence encouraged to identify potential pharmacological agents that would be able to induce ferritin expression and examine whether such induction *via* chemical stimulants of ferritin would provide the same inhibitory effects. To this end we examined the compound D3T that is known to have anti‐oxidant and cancer chemopreventive properties. We demonstrate that D3T‐induced up‐regulation of FtH provides inhibitory effects against high Pi‐ and calcitriol‐mediated osteoblastic differentiation of SMC. It should, however, be noted that D3T is not currently approved for human use. However, it occurs naturally in cruciferous vegetables. The oral administration of D3T is being considered for development as a potential drug for the chemoprevention of hepatic and other carcinomas by the National Cancer Institute.

In conclusion, this study corroborates our previous findings and validates FtH induction as a potent inhibitor of osteoblastic transformation of SMC. Given legitimate concerns regarding utilization of excessive parenteral iron that may potentiate in excessive reactive oxygen species formation, chemical induction of FtH *via* D3T may be a novel preventive measure against vascular calcification. In support of this premise, further studies to test this hypothesis in relevant animal models of vascular calcification is germane and timely given current lack of reliable preventive and/or therapeutic modalities against vascular calcification.

## Conflicts of interest

The authors declare no conflicts of interest.
